# Cross interactions between Apolipoprotein E and amyloid proteins in neurodegenerative diseases

**DOI:** 10.1016/j.csbj.2023.01.022

**Published:** 2023-01-20

**Authors:** Rolf Antonie Loch, Hongzhi Wang, Alex Perálvarez-Marín, Philipp Berger, Henrietta Nielsen, Angeliki Chroni, Jinghui Luo

**Affiliations:** a Department of Biology and Chemistry, Paul Scherrer Institute, 5232 Villigen, Switzerland; b Biophysics Unit, Department of Biochemistry and Molecular Biology, School of Medicine, Institut de Neurociències, Universitat Autònoma de Barcelona, 08193 Cerdanyola del Vallés, Catalonia, Spain; c Institute of Biosciences and Applications, National Center for Scientific Research 'Demokritos', 15341 Athens, Greece; d Department of Biochemistry and Biophysics, Stockholm University, Svante Arrhenius Väg 16B, 10691 Stockholm, Sweden

**Keywords:** ApoE, Amyloid proteins, Interaction, Endogenous molecules, Cytotoxicity, Therapeutics

## Abstract

Three common Apolipoprotein E isoforms, ApoE2, ApoE3, and ApoE4, are key regulators of lipid homeostasis, among other functions. Apolipoprotein E can interact with amyloid proteins. The isoforms differ by one or two residues at positions 112 and 158, and possess distinct structural conformations and functions, leading to isoform-specific roles in amyloid-based neurodegenerative diseases. Over 30 different amyloid proteins have been found to share similar characteristics of structure and toxicity, suggesting a common interactome. The molecular and genetic interactions of ApoE with amyloid proteins have been extensively studied in neurodegenerative diseases, but have not yet been well connected and clarified. Here we summarize essential features of the interactions between ApoE and different amyloid proteins, identify gaps in the understanding of the interactome and propose the general interaction mechanism between ApoE isoforms and amyloid proteins. Perhaps more importantly, this review outlines what we can learn from the interactome of ApoE and amyloid proteins; that is the need to see both ApoE and amyloid proteins as a basis to understand neurodegenerative diseases.

## Graphical Abstract

**Figure:**
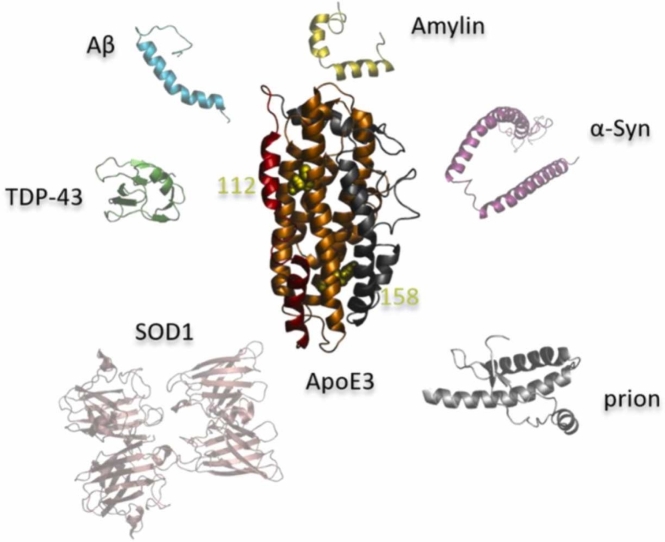

## 1 Background

An increasing number of people suffer from neurodegenerative diseases and other amyloid-based diseases or the amyloidoses, such as Alzheimer’s Disease, Parkinson’s disease, amyotrophic lateral sclerosis, and prion diseases [1]. These lead to immeasurable emotional, social, and economic costs for the patients, their direct environment and societies as a whole. Despite the enormous efforts from academia and industry, no disease-modifying drugs exist and treatment is limited to the management of symptoms or slowing down the progression [2]. These amyloidoses are pathologically manifested by the abnormal deposition of amyloid proteins in cells, tissue or organs, of which the shapes and normal function are altered [1]. There are around 30 different types of amyloid-based diseases, which can be classified into localized and systemic forms, including various peripheral and central variations of these disorders, and many with different subgroups within each disease [1]. It has been suggested that the diversity in normal functions of the related native proteins requires highly dynamic structures that comes at the cost of a higher risk to cleavage and misfolding, which is even further increased by protein mutations and post-translational modifications [3].

The basic structure of all amyloids is the fibril, which is built up by twisted protofilaments that are stacks of protein layers in a β-sheet structure [4]. Amyloid proteins can convert stepwise from soluble monomers into oligomers and then into the insoluble β-sheet fibrils [5]. There are several functional and pathological amyloids. In pathology, amyloids manifest the diseases, in which oligomers may have the most toxic properties [6], even though in nature oligomeric proteins are also the most common structural form of functional proteins [7], [8]. Some amyloid oligomers increase the permeability of cellular membranes that leads to cellular dysfunctions [6]. Oligomers can also trigger other toxic pathways, like mitochondrial dysfunction, synaptic dysregulation and oxidative stress [6], [9]. However, some disease-associated amyloid proteins like Aβ can, sometimes, display protective functions, depending on their location, duration and concentration and the microscopic environmental conditions [10], [11]. This suggests that amyloid proteins can be both protective and damaging, and amyloid homeostasis requires tight regulation which involves the production, post-translational maintenance, aggregation, degradation and clearance [9]. This also means we need to understand better how protein homeostasis, or proteostasis, is maintained and how it is disturbed and which factors can be modified.

The study of amyloid aggregates and their roles in diseases proved extremely difficult and much of our understanding comes from in vitro studies performed under non-physiological conditions. The formation and function of fibrils in vivo can differ profoundly from in vitro fibrils formed from the same recombinant protein [4], while studying the metastable oligomeric intermediates is even more challenging [6], [11]. In addition, misfolding and aggregation can not only be seeded by the same precursor molecules but also cross-seeded by different protein monomers. This can alter the kinetics and toxicity, possibly resulting in diseases with “mixed” pathologies of increased severity and faster progression, and in other cases modulate or inhibit amyloid aggregation and fibrillization or toxicity [12], [13], [14]. Moreover, it is now accepted that all amyloid deposits consist of the key fibril protein, their fragments, and several other components, for example, serum amyloid P-component (SAP) and heparin sulfate proteoglycan [4], which may or may not interact with the amyloid proteins, plus possible co-aggregates with other amyloid proteins [12], [13]. This makes it harder to extrapolate conclusions from isolated in vitro studies to physiological manifestations. This also can explain to a large extent why none of the developed drugs that target single amyloid proteins have been successful so far in curing amyloid-based diseases safely and effectively, although a few may have the potential to slow down the progression when used in the early phases of the diseases [15].

It is now understood that there are many factors and multiple molecules and pathways involved in the progression of diseases, including pro-inflammatory and anti-inflammatory [16]. Likewise, many amyloid diseases can be caused by multiple risk factors, such as multiple genetic, environmental and lifestyle factors. One of the most important genetically related compounds that can interact with amyloid proteins is Apolipoprotein E (ApoE). ApoE is a key regulator of lipid homeostasis and the principal cholesterol carrier in the brain [17]. It is well known for its influence on Alzheimer's and cardiovascular diseases [18]. It has also been identified as one of the two most robust gene loci for longevity [19], and therefore ApoE may be a crucial factor in many other diseases.

**Fig. 1:**
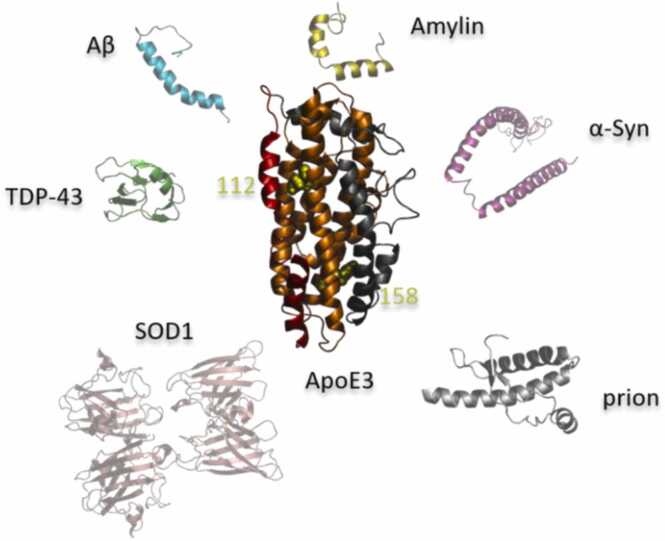
Schematic illustration of the 3D molecular structures of the ApoE 3 structure (pdb ID: 2L7b) and the most studied amyloid proteins that can interact with each other: Aβ (pdb ID: 1BA4), prion protein (pdb ID: 1QLX), superoxide dismutase 1 (SOD1) (pdb ID: 3ECU), α-syn protein (pdb ID: 1XQ8), Islet amyloid polypeptide precursor (Amylin) (pdb ID: 2L86), TAR DNA binding protein 43 (TDP-43) (pdb ID: 5X4F). The residues 112 and 158 distinguish the ApoE isoforms and are shown in yellow spheres. ApoE2 has Cys112 and Cys158 residues; ApoE3 has Cys112 and Arg158 residues; and ApoE4 has Arg112 and Arg158 residues. The N-terminal domain (orange) and C-terminal domain (gray) of ApoE are connected by a hinge domain (red ribbon).

In particular, ApoE polymorphism has a profound effect on multiple molecular and cellular functions in the human body, some of which we will discuss in this paper. There are two kinds of ApoE polymorphism, one genetically determined and one not [18]. Sialyated isoforms are nongenetic post-translational forms with an additional negative charge and account for 10–20 % of plasma ApoE [20]. There are three common genetic Apolipoprotein E isoforms (ApoE2, ApoE3, and ApoE4) expressed by three alleles (ɛ2, ɛ3, and ɛ4) that allow for six phenotypes in humans: three homozygous phenotypes (E4/4, E3/3, and E2/2) and three heterozygous phenotypes (E4/3, E3/2, and E4/2) [18]. ε4, ε3, and ε2 represent the human population of about 14 %, 78 %, and 8 %, respectively [21], and show a geographical variation [21]. ApoE ε4 is considered to be the ancestral allele [19]. The evolution of this allele to other alleles is presumed due to varying environmental and lifestyle conditions that lead to adaptation of diet and altered susceptibility to infections [22]. Within ApoE, a low-density lipoprotein receptors (LDLR)-binding site is located at the N-terminal domain, connected by the hinge to the C-terminal domain with a lipid-binding site (see also Fig. 1 for a schematic representation). The three common ApoE isoforms differ from each other at only two residues: 112 and 158 (ApoE2: 112Cys/158Cys, ApoE3: 112Cys/158Arg, ApoE4: 112Arg/158Arg) [22], [17]. Even though multiple interactions between the N- and C-terminal domains are found to be the same for all apoE isoforms, the single Cys/Arg interchange at sites 112 and 158 does seem to slightly influence the interaction between the two domains interaction in ApoE. The unique intrinsically disordered regions formed by hydrogen-bonds and salt-bridges in ApoE4 [23], [17] have been shown also to occur in ApoE3, and these hydrogen-bonds and salt-bridges have been suggested to be critical for the reversibility of the lipoprotein-binding activity of ApoE [23], [24]. Moreover, it was also suggested that the Cys/Arg interchange affects the lipid binding and lipidation state (lipid-free, partially lipidated or lipid-rich) of ApoE, which is a more important factor in the domain movement, binding affinity, kinetics, receptor interactions and consequently in the multiple functionality of ApoE [23], [24]. For example, the stability of the N-terminal domain is isoform-dependent (ApoE2 > ApoE3 > ApoE4), and ApoE4 has the greatest tendency to form a molten globule state (ApoE4 >>ApoE3 > ApoE2) [17]. This results in profound differences in structure and function, and consequently affects the disease risks in carriers. Furthermore, apolipoproteins possess amphipathic α-helices, which are optimised for the functional (and protective) lipid surface binding, however, when unbound to lipids, this highly dynamic region also has a low thermodynamic stability and high proteolysis, molten globular and amyloid-forming propensity [3]. The ApoE protein is considered a major risk factor for some amyloid-related diseases, including Alzheimer’s disease (AD) [21], Parkinson’s disease (PD) [25], Transmissible spongiform encephalopathies (TSEs) [26], type 2 diabetes (T2D) [27].

In more details, ApoE is a main component of lipoproteins in plasma and the predominant apolipoprotein in the central nervous system (CNS) [28]. Peripheral and central nervous system ApoE are synthesized mainly by liver cells and macrophages, and secreted mainly by astrocytes and microglia [28]. It plays a crucial role in the transportation and metabolism of lipids, cerebrovascular integrity and cerebral blood flow, amyloid clearance, longevity and other functions [19], [21], [29], [30]. ApoE mediates the binding of lipoproteins to ApoE receptors [17]. The ApoE receptors include heparin sulfate proteoglycans (HSPGs), low-density lipoprotein receptors (LDLR), and LDL-receptor related proteins (LRP), with the latter two belonging to the LDLR family [17], [23].

ApoE3 is linked to ‘normal’ plasma lipid levels because of its high efficiency in promoting the clearance of triglyceride (TG)-rich lipoproteins [31]. ApoE2 is restricted in the clearance of TG-rich lipoprotein remnant particles due to its impaired ability to bind to the LDL receptor [32]. In one study including subjects with either APOEɛ3/ɛ3 or APOEɛ4/ɛ4 genotypes, plasma triglycerides were found to be associated with an ApoE isoform-dependent distribution of monomers, homodimers and heterodimers, showing a difference between AD patients and controls [33]. In the same study, ApoE dimers were only observed in the APOEɛ3-carriers and were associated with total plasma ApoE levels [33]. The 112Arg substitution in ApoE4 modulates the inter-domain interaction, which can increase the affinity of ApoE4 with lipids and ApoE receptors [23]. ApoE4 is less stable and more susceptible to proteolysis. This proteolysis can lead to the degradation of ApoE4 into small neurotoxic segments, which may eventually interfere with the normal functions of ApoE4 [34]. Compared with ApoE3 and ApoE2, ApoE4 and its proteolytic, neurotoxic C-terminal truncated fragments are stronger associated with dysfunctions. A study conducted with transgenic mice expressing ApoE3 or ApoE4 in neurons or astrocytes showed that the C-terminal-truncated fragments of neuro-specific ApoE4 are related to increased tau phosphorylation [34]. Compared to ApoE2 and ApoE3, ApoE4 more efficiently induces APP transcription and amyloid-β (Aβ) production, facilitates Aβ fibrillation, interferes with cleaning up Aβ, and utilizes more lipid binding [17]. Therefore, ApoE4 is considered ‘worse’ or less protective than ApoE2 and ApoE3 for patients at the risk for neurodegenerative diseases.

Both ApoE and amyloid proteins play important roles in amyloidoses as they form two main risk factors for these diseases Amyloid and ApoE proteins possess intrinsically disordered regions. These disordered regions control structural motions and interactions of these proteins with lipid membranes, and thereby their functionality. Furthermore, amyloid and ApoE proteins interact with each other. These connections between amyloid and ApoE proteins are predominantly associated with amyloid deposition, with ApoE protein being able to directly modulate the structure and nucleation of amyloid proteins. The function and toxicity of ApoE and amyloid proteins like Aβa tau and α-synuclein can be also influenced by their interactions in amyloidoses [35]. Though there are many therapeutic approaches based on targeting the amyloid protein alone [2], no effective therapies have been established so far for these amyloid diseases.

One reason why there is a lack of progress in treatments, comes from the fact that the molecular and genetic cross-interactions between ApoE and various amyloids have not been clearly interpreted. Therefore, the main focus of this review paper is to better understand the cross-interactions between ApoE and different kinds of amyloid proteins, where we will discuss how ApoE interacts with the generation, aggregation, and clearance of amyloid proteins, and how amyloids affect the intracellular fate of ApoE (Section 2), as well as where they meet (Section 3).

## 2 Cross-interactions of ApoE with amyloid proteins

In this section, we discuss specifically the molecular and genetic interactions of ApoE with the most studied amyloid proteins, including Aβ, tau, alpha-synuclein (α-Syn), prion, amylin/Islet amyloid polypeptide (IAPP), superoxide dismutase 1 (SOD1), transactive response DNA-binding protein 43 (TDP-43) and a few others. A schematic illustration of their molecular structures is shown in Fig. 1.

### 2.1 Interactions between ApoE and the Aβ peptide

Alzheimer’s Disease (AD) is the most common form of dementia and the Aβ_42_ and Aβ_40_ peptides are the most abundant components in the amyloid plaques manifested in the brains of AD patients [36]. In cerebral amyloid angiopathy (CAA), Aβ accumulates and deposits on the wall of cerebral vessels. More than 80% of autopsy-confirmed AD cases have some degree of CAA [37], [38]. It is generally accepted that the formation of Aβ oligomers and aggregates (plaques) is an integrated part of AD pathophysiology [39]. These plaques mainly appear as relatively neutral ‘diffuse’ plaques or disruptive ‘neuritic’ plaques that mostly have a fibrillary or dense-cored morphology [40], [41]. However, the total amyloid-plaque load does not always correlate well with cognitive impairment [42]. This might be due to the difference in initial inflammatory responses between different case groups, especially the response induced by activated glial cells [43]. Nonetheless, the misfolding of Aβ proteins is a key feature in AD and CAA and is impacted by the type of ApoE lipoproteins, as discussed next.

The inheritance of ApoE ε4 is related to late-onset familial and sporadic AD [43], and ApoE ε4 increases the risk of developing CAA and AD, while ApoE ε2 lowers the risk of frontotemporal dementia (FTD) in a very small cohort [44]. ApoE ε2 appears to act as a protective factor against AD and Lewy bodies (DLB) [44]. Reiman et al. found that among the six genotypes of three alleles, ApoE2/2 is related to a low odds ratios of Alzheimer’s dementia in comparison to other genotypes, while ApoE4/4 has the highest odds [45]. Additionally, in a case report, the ApoE3 Christchurch homozygote was reported to resist autosomal major Alzheimer’s disease [46]. With ApoE ε4 as a main genetic risk factor for AD, the cross-interaction between ApoE and Aβ has a major contribution to the progression of CAA and AD.

ApoE is one of many proteins that can interact with Aβ, and the interaction of ApoE with Aβ can affect various stages of Aβ homeostasis as discussed below. However, the binding strength/affinity of ApoE/Aβ complexes varies depending on many factors such as the isoforms, lipidation state, oxidation and concentration of ApoE, as well as the length, concentration, morphology and source (native or synthetic) of Aβ, in addition to the local environmental conditions like receptor concentrations, chaperone concentration, metal ions and electrolyte concentration, pH, temperature, molecular crowding, inflammasome, and other factors that can affect proteostasis [47], [48], [49]. For instance, recombinant ApoE2, E3 and E4 preferably bind to Aβ peptides containing more β-sheet structures with a binding affinity of 20 nM in vitro [50], and lipid-free ApoE binds stronger to immobilized Aβ than lipidated ApoE does [51]. Dissimilarities in these stringent conditions, especially the extent of ApoE lipidation and posttranslational modifications, can, to a large extent, explain the seemingly substantially contradictory results from in vitro and in vivo studies. This is because different lipid-binding states can lead to considerable conformational differences in ApoE and the presence of lipids can alter the binding properties of ApoE with other proteins, like Aβ [52].

**Fig. 2:**
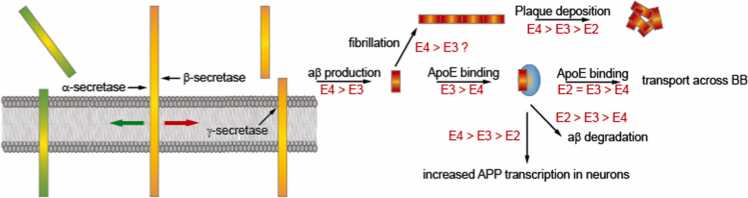
Roles of ApoE isoforms in the processing of APP and Aβ regulations [78]: APP is initially cleaved by α-secretase in the extracellular part near the plasma membrane to generate a soluble protein and a membrane standing part. Metalloprotease of the ADAM family (e.g. ADAM10) possesses α-secretase activity. This pathway is also known as a beneficial pathway. APP can also be cleaved by β-secretase and γ-secretase leading to Aβ and soluble intra- and extracellular proteins (amyloidogenic pathway). β-secretase is an aspartic acid protease (also known as BACE1). γ-secretase is an integral membrane protein composed of PSEN1 (presenilin-1), nicastrin, APH-1 (anterior pharynx-defective 1), and PEN-2 (presenilin enhancer 2). The influence of ApoE isoforms in further processing Aβ is shown on the right side (see main text for details).

We will further discuss how ApoE affects the metabolism, aggregation, degradation and clearance of Aβ peptides. The production of Aβ involves a series of sequential endoproteolytic cleavage by β- and γ-secretases (schematically illustrated in Fig. 2) of the transmembrane protein, amyloid precursor protein (APP), through an anti-trophic amyloidogenic pathway [53]. The influence of ApoE on Aβ production remains controversial and seems to be strongly dependent on the experimental conditions [37]. Despite the fact that several studies [37] reported no apparent ApoE isoform-dependent effect on APP, one in vitro study conducted with rat neuroblastoma B103 cells transfected with human wild-type APP695 (B103-APP) [54] proposed that lipid-poor ApoE4 enhances higher Aβ production from APP compared with lipid-poor ApoE3, which was mediated through the low density lipoprotein receptor-related protein (LRP) pathway. In another in vitro study using human neurons induced from embryonic stem cells and in vivo experiments in mice [55], ApoE differentially (ApoE4 > ApoE3 > ApoE2) stimulates APP transcription and Aβ secretion by triggering a non-canonical MAP kinase cascade by binding to ApoE receptors. In addition, a recent study suggested that reconstituted ApoE isoforms increase BACE1 levels and promote Aβ production and oligomerization in human neuroblastoma SK-NSH cells with distinct efficiency (ApoE4 ≥ ApoE3 > ApoE2) [56]. The cleavage of APP by γ-secretase and then the production of the APP intracellular domain (AICD) in the CNS can attenuate the expression and function of lipoprotein receptor (LRP1) [57]. A reduced LRP1 level leads to an increased ApoE level that could further increase APP transcription as mentioned above, although reduced LRP1 also reduces the amyloidogenic processing of APP. Meanwhile, a lower LRP1 level can also decrease the cholesterol level within the CNS, and result in the loss of neuronal membrane cholesterol that was also found to enhance the Aβ production, suggesting a feed-forward loop [57]. The combined results can explain to a certain degree the relationship between APP, ApoE, and AD.

As for the effect of ApoE on Aβ aggregation, substoichiometric concentrations of ApoE dramatically inhibit Aβ fibril growth via binding to and stabilizing oligomers, while higher concentrations of ApoE can also bind to and stabilize Aβ fibrils directly [58]. In addition, ApoE4 is the most efficient in influencing the fibrillation of Aβ40 while ApoE3 and ApoE2 function less efficiently [58]. Besides, ApoE3 binds preferentially to Aβ42 oligomers, leading to the inhibition of the Aβ42-induced neurotoxicity, which has been shown to be ApoE-isoform dependent (ApoE3 > ApoE4) [59]. This isoform-dependent effect of ApoE might arise from the differential binding affinities of the ApoE isoforms to different aggregation intermediates or from the altered lipidation states of ApoE in the brain, which is regulated by ATP binding cassette transporter A1 (ABCA1) [47]. Thus, the destructive role of ApoE4 in AD could be partly due to the stabilization of the soluble cytotoxic oligomeric intermediates, as well as the insoluble fibrils. Fragments of ApoE and other apolipoproteins, such as ApoA-I (the major compound in HDL) and Apo-J, may also play simultaneously a protective and toxic role in Aβ aggregation [60], [61].

In addition to the effects of ApoE, especially ApoE4, on the kinetics of Aβ oligomerization and fibrillization process, Aβ in turn also affects the oligomerization, aggregation as well as neurotoxicity of ApoE4. When co-incubated in vitro in human neuroblastoma SK-N-SH cells, Aβ42 increases the formation of SDS-stable ApoE4 oligomers and the cytotoxicity of ApoE4 [62]. These data highlight that ApoE4 could be pathogenic due to its capacity of oligomer assembling that is increased by Aβ42 and leads to higher neurotoxicity.

ApoE and its fragments can also positively or negatively affect the degradation and clearance processes of Aβ, depending on the lipidation state of ApoE [63], [64]. The promoting effect of ApoE on the cellular degradation of Aβ has been reported by in vitro studies [65], and the impaired degradation may subsequently deteriorate further clearance, or the other way around. There are several mechanisms underlying the clearance of Aβ from the brain. Of these, the receptor-mediated clearance via microglia/astrocytes or through the blood-brain barrier (BBB), and the clearance through the interstitial fluid (ISF) drainage play the dominant roles [66]. ApoE affects the Aβ clearance in different ways and is dependent on several factors. Different Aβ levels can be transported across the BBB in an ApoE-isoform and -lipidation state dependent manner [67]. *In vitro*, human astrocytes preferably take up oligomeric Aβ over fibrillar Aβ [68], while human microglia more efficiently take up fibrillar Aβ compared with astrocytes[69]. These uptake processes in microglia and astrocytes are negatively affected by ApoE and ApoJ [69]. ApoE receptor-mediated clearance of Aβ generally occurs in microglia and astrocytes, where soluble Aβ can bind directly to LRP1 of astrocytes in competition with ApoE isoforms [66], [70]. Mouse studies suggest that the clearance of Aβ from ISF into the blood can be facilitated by p-glycoprotein (Pgp) and LPR1 expressed by the vascular endothelial cells [71], [72], [73]. However, ApoE4, compared to ApoE2 and ApoE3, shows a relatively low affinity for LRP1 and is therefore less efficient in promoting Aβ excretion through the BBB [30], [67], [71]. Furthermore, APOE is critical for maintaining cerebrovascular integrity, as ApoE-deficient mice show more vascular defects and the BBB is broken down more, even before neuronal and synaptic changes occur, although these effects occur in ApoE4 mice. Thus it seems that ApoE4 is less protective than ApoE3 and ApoE2, and the BBB of ApoE4 is consequently more susceptible to, for example, injury [30]. This is consistent with the observation that (in mice) ApoE2, ApoE3, Aβ/ApoE2 and Aβ/ApoE3 complexes were cleared at the BBB via both LRP1 and VLDLR (very low density lipoprotein receptors) at a faster rate than Aβ/ApoE4 complexes that was cleared mainly via VLDLR alone [67]. In addition, lipidation slowed down the clearance of all the ApoE isoforms and ApoE/Aβ complexes at the BBB by a factor of 2–3 [67]. On the contrary, ApoE4-containing lipoproteins are less lipidated than ApoE3 [47], which might lead to the less stable ApoE4/Aβ complexes, resulting in reduced ApoE4/Aβ levels, decreased clearance, and increased accumulation of Aβ, especially in oligomeric form. An additional degree of complication in the interaction of ApoE/Aβ and LRP1 is that the significantly reduced LRP1 levels in the vascular endothelial cells and pericytes of AD patients are related to a noticeable decrease in the ApoE-dependent removal of soluble [71], [72], [73]. This is consistent with the above-mentioned observation that the formation of Aβ is accompanied by the APP processing product, the APP intracellular domain that suppresses the expression of LRP1. Alternatively, Zekonyte et al. proposed that the efficiency of perivascular clearance of Aβ is reduced by the presence of ApoE4 and enhanced in the presence of ApoE3 [74]. This is because the attachment of ApoE4/Aβ complexes to the arterial basement membranes is weaker than that of ApoE3/Aβ complexes [74]. In addition, the attachment of ApoE/Aβ complexes to the arterial basement membranes again strongly depends on the type of Aβ and the ApoE lipidation status [47], which may result from the strong destabilization of the salt bridge network of ApoE4 by Aβ [75]. As a result of these many interactions, compared with ApoE2 and ApoE3, ApoE4 correlates with higher Aβ levels in cerebrospinal fluid (CSF) and cerebral vasculature. This can be used as a biomarker in the preclinical phase of AD.

Despite the fact that the ApoE/Aβ interaction has been investigated in vitro, the direct molecular interaction between ApoE and soluble Aβ under physiological conditions has been shown to be minimal: the uptake and subsequent degradation of Aβ in astrocytes are blocked by ApoE isoforms that compete for the same cellular receptors, transporters, and/or cellular/membrane surfaces, especially the LRP1-dependent cellular uptake pathway in astrocytes. Thus, the role of ApoE in the clearance of Aβ from CNS is highly complex and remains yet to be resolved and may be profoundly different in vivo from in vitro. However, this controversy has not prevented the use of ApoE-derived peptides and ApoE/Aβ antibodies as therapeutic agents for AD treatment [76], [77]. Interpreting the results of all these studies combined, we can say little more than that for healthy functioning and homeostasis there exist an optimal concentration and an optimal level of lipidation of ApoE, which appear to be isoform dependent.

### 2.2 Interactions between ApoE and Alzheimer’s disease-associated tau

Tau is mainly a neuronal protein existing in axons and is mostly associated with the stabilization of microtubules therein. Tau can be modified by phosphorylation, the state of which will in turn influence the distribution of it. Tau tends to be phosphorylated in the cytosol while dephosphorylated in the distal region of the axon [79]. Furthermore, the phosphorylation state differs according to the development stages of tauopathic diseases and healthy states. The extent of phosphorylation is higher in fetal neurons and decreases during the development period, but increases hugely in pathological situations (tauopathies), which may be related to the normal and abnormal functions of tau [80]. Under normal physiology conditions, tau binds to microtubules, as well as lipid membranes, to maintain the normal shape and function of neurons. However, under pathological conditions, tau phosphorylation is high, leading to the reduced affinity for microtubules and subsequently cytoskeleton destabilization, particularly in neurons. The detached, hyperphosphorylated tau then undergoes self-aggregation, forming oligomers and various higher-ordered aggregates, which are involved in AD and some other tauopathies [79]. In fact, AD patients usually have more hyperphosphorylated brain tau [79]. Mild cognitive impairment (MCI) patients who are ApoE4 carriers also have high levels of CSF tau and bear the elevated risk for AD [81]. However, CSF ApoE levels do not show a consistent association with AD and MCI among different studies, although CSF ApoE levels might be more closely correlated with tau, phosphorylated tau, and Aβ42 in AD and MCI patients than plasma ApoE levels [82]. In addition, it has been reported that ApoE4 significantly aggravated tau-mediated neurodegeneration in a tauopathy mouse model [83]. In an earlier study, a C-terminal truncated ApoE form, the ApoE4 [Δ(272–299)] in neurons, was found to induce the intracellular neurofibrillary tangles NFT-like inclusions via interacting with neuronal phosphorylated tau as well as phosphorylated neurofilaments [34], leading to mitochondrial dysfunction and a 35 % greater cell death than full-length ApoE4 [84]. These studies indicate the role of ApoE in influencing tau pathogenesis, in which ApoE-induced neuroinflammation is involved.

As a major genetic risk factor for AD, ApoE exerts an isoform-specific effect on the hyperphosphorylation of tau and the neuronal cytoskeleton. ApoE3 binds in vitro stably to the microtubule-binding repeat region of tau, thus stabilizing it and inhibiting the formation of paired helical filaments (PHF) [85]. This interaction can be inhibited by the phosphorylation of tau [86], suggesting that ApoE3 may protect tau by preventing hyperphosphorylation in vivo [85]. In contrast to the protective role of ApoE3, ApoE4 shows reduced binding to tau. This may signify a loss of protective function when stimulating tau phosphorylation in a neuron-specific way, resulting in higher amounts of unbound tau in the CSF [87], [88]. This is consistent with the observation in AD and MCI patients as mentioned above.

In vitro studies show that ApoE regulates tau phosphorylation partly due to the modulation of the activities of tau phosphatases and kinases. Dephosphorylation of (synthetic) tau was the result of ApoE affecting Ca^2+^-associated signal transduction pathways that enhance the activity of protein phosphatases 2A/2B (PP2A/2B) [89]. This effect is more pronounced in ApoE3 and ApoE2 than in ApoE4. Oppositely, ApoE4 enhances tau phosphorylation by promoting the activation of zinc-induced extracellular signal-regulated kinase (Erk) in neurons [90], while there is also a reduction in PP2A activity by ApoE4 compared with ApoE3 via epigenetic mechanisms [91]. Another study suggested that ApoE controls tau phosphorylation by the assembly of the ApoE receptor/disabled-1/glycogen synthase kinase-3β (RAD) complex, which in turn suppresses the kinase activity [92]. Furthermore, ApoE-deficient mice have hyperphosphorylated tau in the brain compared to wild-type controls, and show diminished repair after a closed head injury that is related to transient hyperphosphorylation [93]. Interestingly, some researchers even argued that only at a high concentration (2 μM), ApoE reduced the levels of tau kinases and then the levels of tau phosphorylation in primary neurons [94]. This concentration dependency of ApoE on tau illustrates that there can be significant differences in ApoE-tau interactions under in vivo and in vitro conditions which have to be taken into account. It also suggests that there is an optimal concentration of ApoE to regulate phosphorylation that is isoform dependent. However, there is insufficient data available on the effect of the lipidation state of ApoE and tau phosphorylation, or any other posttranslational modifications of ApoE.

In contrast to the effect of ApoE on tau, tau also influences the intracellular fate of ApoE in an ApoE isoform-specific manner. When transiently expressing tau and LDLR in mammalian cells, ApoE is taken up from the CSF, but only ApoE3, not ApoE4, reaches the cytoplasm [72]. Another study showed that especially non-lipidated ApoE2 could form a complex with tau in-vitro [69]. This highlights the isoform- and lipidation-dependent cross talk between ApoE and tau.

In summary, the phosphorylation state of tau plays crucial roles under both physiological and pathological conditions. ApoE influences the phosphorylation state of tau in an isoform-specific way, with ApoE3 binding stably to tau while ApoE4 binding less efficiently. This results in the inhibitory effect of ApoE3 and promoting effect of ApoE4 on tau phosphorylation, influencing the cytoskeleton stability and the formation of NFTs in AD.

### 2.3 Interactions between ApoE and Parkinson’s disease-related α-Syn

α-synuclein (α-Syn) is mainly a neuronal protein in the brain with multiple functions, and it can also be generated in the intestine and transported through the vagus nerve to the brain [95]. Located primarily in the pre-synapses, α-Syn is involved in the neurotransmitter release by interacting with lipid rafts. This interaction may be enhanced by the high curvature of synaptic vesicles and the property of α-Syn to promote and sense membrane curvature. However, overexpression of α-Syn inhibits exocytosis from the synaptic vesicles [95], [96]. Besides the role in the maintenance of synaptic vesicles, α-Syn may also function as a lipid acceptor, since it shares biophysical similarities with other lipid-carrying proteins [97].

α-Syn is an intrinsically disordered protein that is prone to aggregate into intracellular inclusions [98]. Overexpression and misfolding of α-Syn as well as mutations within the α-Syn gene ( SNCA) have been linked to Parkinson’s disease (PD), characterized by Lewy bodies (LB) [98], [99].

Even though it has been shown that ApoE4 exacerbates α-Syn pathology [25], the role of ApoE on PD pathogenesis remains to be further explored. The level of ApoE in CSF is higher in patients with early PD, and α-Syn was observed to colocalize with ApoE on lipoprotein particles [100]. In addition, the frequency of ApoE ε4 carriers is higher in both PD patients with dementia (PDD) than that in healthy controls [101]. And among PD patients, the frequency of ApoE4 carriers is higher in familial PD than that in sporadic PD [102]. Although not every person with ApoE4 develops PD and many PD patients possess one of the other alleles, there appears an increased risk for ApoE ε4 carriers to develop PD and they might have an earlier age of the disease onset as compared to ApoE ε3 carriers. In addition, PD patients carrying ApoE ε4 might have a greater risk to develop additional dementia, in comparison to non-ApoE4-carriers. Contrary to AD, in PD patients the ApoE ε2 allele frequency is also higher than that in controls, and thus ApoE ε2 allele can also be considered as a risk factor for developing PD [103], [104]. This was attributed to a disturbance of the ApoE homeostasis and then the subsequent dysfunction of the protein.

As mentioned above, α-Syn acts as a lipid binding acceptor and, thus, the cross talks between α-Syn, ApoE, lipoproteins, and lipids are relevant because they mutually influence both lipid (especially cholesterol) metabolism and α-Syn properties such as the uptake and the tendency for aggregation [105], [106]. These cross talks heavily depend on several other factors like the concentration and lipidation state of ApoE as well. Namely, it has been reported that α-Syn strongly stimulates the outflow of cholesterol efflux [107], while a higher (optimal) level of plasma cholesterol is related to a lower risk of PD [108], [109]. In both incidental LB disease (iLBD) and symptomatic stages of PD, the up-regulation of ApoE and LRP1 in the melanized neurons of the substantia nigra (SN) is associated with alterations in lipoprotein homeostasis, presumably as an early event in the process of PD development [110], possibly in an attempt to prevent aggregation of α-Syn. An in vitro study showed that ApoE influences the aggregation of α-Syn in a concentration-dependent way [107]: at a low concentration of 15 nM, all three ApoE isoforms promote the aggregation of α-Syn where ApoE4 shows the strongest efficacy. At the higher concentrations (> 15 nM), the isoforms suppress the aggregation of α-Syn. This study suggests that the concentration and isoforms of ApoE significantly influence the kinetics of α-Syn and thus ApoE can play both protective and toxic roles. However, how this converts to in vivo concentrations is unclear. A molecular link between α-Syn, ApoE and Aβ has also been revealed in a study with transgenic mice expressing either wild type or mutant α-Syn [111]. It was shown that increased levels of ApoE are injurious, leading to α-Syn-induced neurodegeneration, as well as the elevated accumulation of insoluble mouse Aβ. This study indicates a common pathogenic mechanism of ApoE in various neurodegenerative diseases, including AD and PD. Although detailed mechanisms are still lacking, through the cross interaction between ApoE, α-syn, and lipids (especially cholesterol), we can say that ApoE influences the uptake and aggregation of α-Syn as well as the lipid metabolism, and there seems to be an optimal level for ApoE since both a too low and a too high concentration is associated with increased amyloid aggregation.

### 2.4 Interactions between ApoE and TSE-related prion

The aggregation of the pathological isoform of the prion protein (PrP) in the CNS is the main hallmark of a group of neurodegenerative disorders termed Transmissible spongiform encephalopathies (TSEs), including Creutzfeldt–Jakob disease (CJD) [112]. The ApoE level is higher in the sporadic Creutzfeldt-Jakob disease (sCJD) cases [113]. ApoE co-occurs and co-deposits with PrP in TSE infected brains [114]. Furthermore, ApoE was co-purified with PrP identified by proteomic analysis[112], [113]. Biophysical and biochemical studies revealed that the recombinant PrP protein interacts with both recombinant ApoE and native ApoE from hamster liver tissue in vitro [115]. This ApoE-PrP interaction has been mapped to the N-terminus of both proteins, namely, the residues 1–194 of ApoE and the residues 23–90 of PrP [115]. Aβ42 and ApoE were identified in the majority of the sCJD [113] and the ε4 allele is a risk factor for CJD, while ε2 delays the occurrence of death [26]. A meta-analysis showed no significant evidence between ApoE2 and CJD, although the ApoE 3/4 and ApoE 4/4 genotypes are risk factors for CJD, while ApoE 3/3 genotype may protect against CJD [116], [117]. A synergistic age-dependent interaction in AD and sCJD has been observed between ApoE and the prion protein gene (PRNP) [118]. A recent study suggested that AD/primary age-related tauopathy (AD/PART) and CJD pathology can occur in the same brain [119]. Still, ApoE correlated only to Aβ pathology and did not show to influence the age of onset and subtypes of CJD, indicating that the synergistic mechanisms of AD and CJD remain to be better understood. The findings of the few aforementioned studies suggest that ApoE4 does play a predominant role in TSEs but the exact mechanisms remain to be further clarified.

### 2.5 Interactions between ApoE and T2D-associated IAPP

Amylin, also called Islet amyloid polypeptide (IAPP), is cleaved from the pro-islet amyloid polypeptide (proIAPP) and co-secreted with insulin from the pancreatic β-cells. IAPP, on the one hand, plays various functional roles physiologically (in glucose homeostasis, glucagon release, gastric emptying and antimicrobial activity [120], [121]). On the other hand, it can transform from soluble, functional states into highly organized amyloid deposits that contribute to the death of pancreatic β-cells and thus act as one of the most important hallmarks of type 2 diabetes (T2D), together with insulin resistance and deficiency [120], [121], [122], [123], [124], [125]. It is worth mentioning that lifestyle and environmental factors are accepted as the main causes for developing T2D [126], [123], while T2D doubles the risk for developing dementia and AD, and a subgroup among AD associated with insulin resistance has been dubbed type 3 diabetes [124]. In addition to the aforementioned cross interactions between Aβ, tau, α-Syn, or prion and ApoE, an association between ApoE and IAPP also exists. In particular, the colocalization of ApoE and IAPP suggests that ApoE plays an important role in the development of multiple amyloid deposits [127]. However, it remains to be clarified whether the role is protective, toxic or a loss of protection.

ApoE was found not to be crucial for the formation of IAPP amyloid fibrils in a transgenic mouse model of T2D [128], unlike the case in AD. However, a clinical study with T2D patients from China indicated that ApoE ε4 enhances islet amyloidosis [129]. Furthermore, it has been shown in human samples and AD mouse models that ApoE4 attenuates the IAPP-assisted clearance of Aβ40 from the AD brain into blood [130], suggesting the risk factor of IAPP and ApoE4 interaction in AD. The effect of ApoE on T2D may be related to the modulation of IAPP aggregation in a concentration-dependent manner. ApoE and the incompletely processed proIAPP promote the fibrillation of IAPP and coexist with the amyloid [120]. The fibrillation of IAPP can be inhibited at a low concentration of ApoE4 but is promoted at a high concentration of ApoE4 [131]. This implies that under healthy physiological conditions ApoE4 may prevent IAPP aggregation by efficiently binding and sequestering IAPP, however, the enhanced binding affinity between ApoE4 and IAPP in T2D results in the critical accumulation of IAPP and subsequent islet amyloid formation [132]. Like the interaction between ApoE and Aβ [50], the interaction between ApoE and IAPP partially depends on the state of ApoE (concentration and lipidation). These studies suggest again that the cross interactions of ApoE and amyloid proteins presumably rely on specific (largely unknown) microscopic conditions that differ between healthy and sick people. In addition, cholesterol homeostasis is strongly related to ApoE, and recent studies suggest that gangliosides and cholesterol affect hIAPP-membrane interaction and the uptake and clearance of hIAPP [133], [134]. This implies an indirect role of ApoE in IAPP clearance, which requires further investigation. Further research is also required to understand investigation remains to be understood for the indirect effect of impaired ApoE functionality, stability and degradation due to isoform-specific (apoE2 > apoE4 > apoE3) glycation in hyperglycemic diabetic patients [133], [134].

### 2.6 Interactions between ApoE and ALS-related SOD1

SOD1 is an important antioxidant enzyme that can internalize the superoxide (O2−) into relatively nontoxic molecules [135]. There are three known forms of SOD in humans, in which mutations in the gene encoding copper, zinc superoxide dismutase 1 (Cu, Zn-SOD1) are associated with amyotrophic lateral sclerosis (ALS) [136]. ALS is a common motor neuron disease, and mutations in SOD1 gene are present in 2–6 % of all ALS patients [136].

It has been reported that ApoE is also involved in ALS [137]. In familial ALS-linked transgenic mice, ApoE expression is strongly upregulated in the spinal cord of end-stage disease mice with SOD1 (G93A-SOD1) mutation [137]. This upregulation is closely linked to the time and location of both the axonal and neuronal degeneration, as well as the glial activation and the activation of genes in mitochondrial destruction [138], [139]. This is consistent with other studies in which ApoE ε4 allele is associated with earlier onset [137] and reduced life span in ALS patients [140]. This means that ApoE4 is more toxic or less protective in ALS. In agreement with it, ApoE ε2 has been reported to delay the onset for 3 years compared to that of non-ApoE ε2 carriers [141]. Because of the limited number of available studies, the role of ApoE in ALS and the ApoE interaction with SOD1 remains to be further investigated.

### 2.7 Interactions between ApoE and TDP-43

TDP-43 is a 414–amino acid RNA-binding protein involved in several cellular processes [142], [143]. In addition, during cellular stress, TDP-43 enters into the cytoplasm as a component of insoluble aggregates with a ubiquitinated, hyper-phosphorylated and cleaved form of TDP-43 with a higher molecular mass at ∼ 45 kDa [144]. The latter form is the major disease protein in some sporadic and familial ALS, most cases of frontotemporal dementia (FTLD-TDP) [144], and in AD and some PD patients [145], [146], [147]. Moreover, it was shown to be associated with memory loss and progressive hippocampal atrophy [148]. Recently, a strong association was reported between ApoE4 and TDP-43 [149]. In particular, the ApoE4 isoform and TDP-43 appear to synergistically affect the cognitive impairment of AD patients, independent of Aβ. In a study with AD subjects with and without TDP-43 having similar degrees of AD pathology, especially during the early phases of neurodegeneration, while normal cognition was strongly associated with the absence of TDP-43 [150]. TDP-43 was also found to be co-localized with tau, and both amyloids are present in a number of diseases [151].

Furthermore, abnormal levels of TDP-43 were observed in the majority of patients with AD, ALS, and FTLD, and there is a substantial increase (200 %) in TDP-43 levels in cortical autopsies of late stage AD patients [150], [152]. It was suggested that overexpression of TDP-43 might be harmful and induce neurodegeneration, possibly with or without formation of insoluble protein aggregates. The mutant TDP-43 (A315T, G348C and A382T) may cause disease via both loss-of-positive-function and gain-of-toxicity [153]. The loss of functional TDP-43 may disrupt key nuclear functions, resulting in the loss of cellular homeostasis, transcriptional deregulation, disintegration of nuclear bodies, or aberrant messenger RNA splicing [142], [144]. Expression of Aβ42 (independent of tau) was associated with TDP-43 phosphorylation, up-regulation, cleavage and accumulation in the cytosol, while α-Syn does not seem to affect TDP-43 much [152]. TDP-43 oligomers can also cross-seed amyloid oligomers with Aβ [154].

**Fig. 3:**
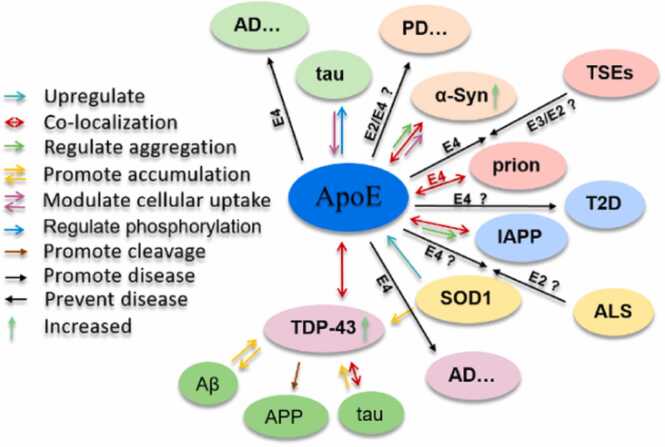
Diagram illustrating the interactions between ApoE and tau, α-Syn, prion, IAPP, SOD1, and TDP-43. For details, please refer to 2.2, 2.3, 2.4, 2.5, 2.6, 2.7. The question marker means that even though most studies agree, a consensus has not been reached in the whole field. Specific disease-related amyloid proteins are labeled with the same color as the labels with their disease abbreviation.

Furthermore, there exists an indirect link between ApoE4, TDP-43 and Aβ. The depletion, aggregation, as well as the disease associated mutation of TDP‐43 can disturb intracellular sorting and subsequent activity-dependent secretion of the neurotrophin brain-derived neurotrophic factor (BDNF) [155]. ApoE4 subjects were found to have significantly lower serum BDNF levels [156]. The interruption of BDNF signaling in hippocampal neurons was found to rapidly activate the amyloidogenic pathway for Aβ production, causing neuronal apoptotic death [53]. ApoE4 subjects were found to have significantly lower serum BDNF levels [132], and a BDNF reduction contributes to lower cognitive performance in both AD apathetic patients and female ApoE4 carriers [157] and non-demented subjects [158]. However, how these processes are linked to ApoE is currently unknown at the molecular level. A schematic summary of the above-mentioned amyloid proteins and their interactions with ApoE is provided in Fig. 3.

### 2.8 Interactions between ApoE and other amyloid proteins

**Fig. 4:**
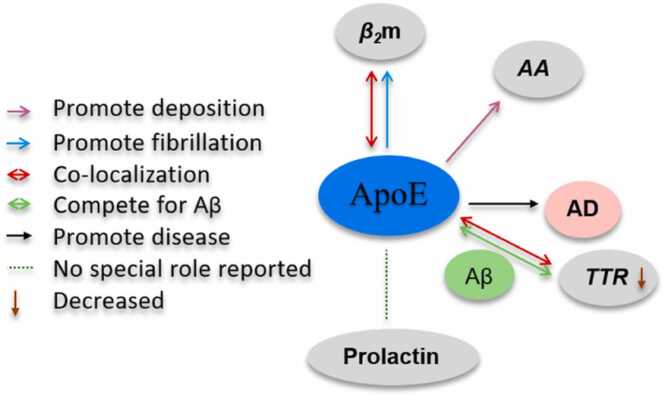
Interactions between ApoE and other amyloid proteins.

In addition to the interactions with the aforementioned amyloid proteins, ApoE also interacts with other amyloids such as transthyretin (TTR), β2-microglobulin (β2m), Prolactin, and Amyloid A (AA), as schematically illustrated in Fig. 4. However, there is much less data available on these interactions, and further research is required to draw any conclusions.

The co-localization of ApoE and TTR has been reported in the systemic organs except for the liver and spleen of an aged male vervet monkey especially in the extracellular stroma among muscle fibers and external tunica of arterioles [159]. Both ApoE and TTR are Aβ sequestering proteins forming a stable compound and affecting the functioning of Aβ. TTR levels in the CSF of AD patients are decreased and even more among ApoE ε4 allele carriers. Reduction in CSF TTR levels leads to decreased levels of CSF Aβ42 and increased levels of senile plaque (SP) abundance in the AD brain [160]. Due to the facts that ApoE induces the accumulation of Aβ and that TTR facilitates the transport of Aβ from the brain and prevents fibril formation [161], ApoE and TTR may act competitively in the aggregation of Aβ and its deposition in the SP of AD brain.

ApoE was also found to be co-located with β2m in amyloid depositions [162]. Furthermore, ApoE can enhance β2m fibrillogenesis processes by stabilizing preformed seeds [163], [164], and then stabilize β2m fibrils by inhibiting its depolymerization [165]. The existence of ApoE induces AA amyloid deposition in a mouse model[166]. Another study employing ApoE-deficient mice with or without injecting adenoviruses expressing the human ApoE3 gene showed that ApoE (ApoE3) promotes the deposition of AA [167].

## 3 Potential interaction mechanism between ApoE isoform and amyloid protein

**Fig. 5:**
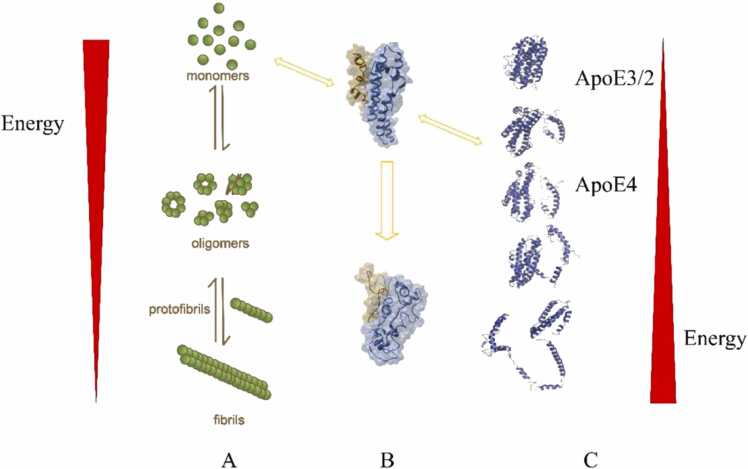
Proposed mechanism of the interaction between amyloid proteins and ApoE isoforms. A, C). In amyloid protein aggregation, monomeric proteins search low-energy states and usually deposit as amyloid fibrils through transit and high-entropy oligomers. Compared to ApoE2 and ApoE3, ApoE4 is likely to fold as a molten globule state where the N- and C-terminal domains unfold with higher entropy. B). Alternatively, both ApoE4 and amyloid protein, with high entropy and co-occurrence in the brain, are prone to cross reaction for seeking a lower entropy and free energy. Due to the higher entropy state, the interaction may be more easily modulated by other factors, like lipidation discussed in this review.

To discriminate the different interaction mechanisms of ApoE isoforms with amyloid proteins, we propose the energy landscapes of ApoE isoform protein folding and amyloid aggregation in Fig. 5. The energy landscapes describe the relative stabilities of different aggregated and folded states. Different energy states are linked to structural, dynamical and functional differences. Intrinsically disordered amyloid proteins have a high degree of entropy and free energy and will transform to lower free energy states by aggregating, usually via transient intermediates. These transient states co-aggregate to each other and form oligomers and then fibrils. This is schematically illustrated in Fig. 5A. In the process of protein folding, the number of conformational states reduces and, hence, the entropy and the free energy as well in Fig. 5**C**. Intramolecular interactions lead to protein folding toward the natively soluble state. This competes with intermolecular interactions in Fig. 5B that can lead to different amyloid states, which can be disordered (amorphous aggregates) or highly ordered (polymorphic amyloid fibrils) and are less soluble due to exposure of hydrophobic amino acid residues.

The three ApoE isoforms, on the other hand, also have multiple intermediate states at different temperatures with varying thermal stability, according to molecular dynamics simulations [168]. ApoE4 has a lower thermal stability as compared to ApoE2 and ApoE3. It suggests that, despite minimal differences in the primary structures, the differences in physiological behaviour among the isoforms can be explained by the difference in folding mechanisms related to the minimal mutations in the amino acid sequences. In particular, all of the isoforms could be characterized by different intermediate states for structural rearrangement upon binding events. They identified a unique, more compact and stable ApoE4 misfolded intermediate state with increased inter-domain interactions. This more stable intermediate state was speculated to change the interaction with lipids and lipoprotein and thereby modify the lipid transport efficiency and affect the kinetics of aggregation and clearance mechanisms of amyloid proteins.

Other studies also suggest that ApoE4 has a higher population of a more chemically and thermally stable intermediate in the form of a flexible molten globule state that exposes the hydrophobic core due to a partial opening of the four-helix bundle [169], [170]. It was also suggested that the open conformation of the intermediate is similar to the conformation change of ApoE once it binds to lipids. These stable intermediate molten globules have nearly native structural features and are responsible for the stronger interdomain FRET signals of ApoE4 in mildly denaturing conditions, in particular at a low pH, and even under native conditions as opposed to ApoE3 [169].

Molten globule states have several physiological roles. They have increased affinity for membranes and bind to liposomes and phospholipids, although they are also known to be prone to misfolding, aggregation, post-translational modifications and proteolytic cleavage [170]. We also agree that ApoE4 molten globules within lysosomes and endosomes may act as a possible route for the commonly observed pathological features in neurodegenerative diseases.

To summarize, besides the higher susceptibility of ApoE4 to form toxic fragments, the formation of a higher amount of more stable intermediate molten globules with ApoE4 could be an important factor to explain the differences in the interactions between ApoE isoforms and amyloid proteins as described in Section 2. Another important factor to take into account, is the loci of cross-interactions, which will briefly be discussed in the next section.

## 4 Loci of cross interactions

### 4.1 Phase-separated droplets

Through phase separation, an emerging paradigm underlies intracellular processes, such as amyloid protein aggregation in neurodegenerative diseases [171]. Phase separation enhances the concentration of amyloid proteins and promotes their self-assembly, typically in membrane-less organelles. For example, full-length tau proteins (tau441-GFP) were observed to form droplets in cultured neurons, with more rapid droplet formation in the presence of heparin and RNA, or under higher temperature or phosphorylation [172]. The C-terminal domain of TDP-43 undergoes phase separation into droplets, with slower dynamics or faster aggregation in all disease mutants [173]. A series of other amyloid proteins have been found to form droplets summarized in the review paper by Elbaum-Garfinkle [174]. Similar phase-separation droplets, lipid droplets (LDs), surrounded by a layer of lipids and proteins, act as inert cellular reservoirs and are associated with ApoE and neurodegenerative diseases [175]. Farmer et al. found several differences in liquid droplets formation and fatty acid metabolism with astrocytes expressing ApoE4 or ApoE3. Phase-separated droplets or organelles apparently offer ideal mesoscopic locations for cross-interactions of ApoE with amyloid proteins, although how the interactions in the droplets take place remains to be investigated.

### 4.2 Lipid membranes

Playing a key role in lipid metabolism, ApoE binds and redistributes lipids, especially from astrocytes in the CNS to other types of cells, including neurons [176]. Likewise, most amyloid proteins experience conformational changes upon binding lipid membranes and self-propagate through a lipid membrane-enriched environment such as membrane boundaries, cellular compartments and tissues [12], [14], [177] Lipid induced conformational changes of amyloid proteins, on the other hand, modulate amyloid protein aggregation and toxicity, which varies with lipid composition and metal ions [13]. The co-occurrence, accumulation and interaction of amyloid proteins and ApoE in/on lipid membranes or the membrane boundaries seem highly plausible at the microscopic level. The affinity of ApoE and its signal peptide toward membranes depends on the lipid composition of the membrane, and it was shown that the ApoE signal peptide binds more tightly to membranes with low cholesterol concentrations, such as found in the endoplasmic reticulum (ER) [178]. Further research is needed in this direction. The fact that molten globule intermediates have a higher affinity for lipid membranes and that such a state is more readily formed with ApoE4, as discussed above, emphasizes the need for further research in this direction.

### 4.3 Cholesterol and lipidation

The disturbance of lipid-related molecules and biological processes are deeply involved in neurodegenerative diseases, especially AD [179], [180]. There exist an optimum level for cholesterol as both too low and too high cholesterol are recognised risk factors for AD, resulting in pro-inflammatory responses and increased Aβ and tau amyloids, as reviewed by Gupta and Weaver [181]. With molecular dynamics simulations of model neuronal membranes, they showed that too high cholesterol concentrations make the membrane less permeable by compacting and stiffening it, and that too low cholesterol results in compromised membrane integrity via higher molecular disorder and tilting, and increased fluidity and interdigitation of the leaflet lipids [181]. The cholesterol concentration then also affects the lipid rafts and the proteins associated with it [182], which are also the most common modulators for both amyloid and ApoE proteins. Cholesterol is an important constituent of the plasma membrane. As a molecular spacer, it can stabilize lipid rafts by occupying the space between raft proteins and other raft lipids [183]. There is an interplay between raft proteins and raft lipids. On the one hand, the regulation of cholesterol homeostasis can lead to the dissociation, dysregulation, and/or inactivation of raft proteins. On the other hand, raft proteins including amyloids can influence cholesterol homeostasis. The cholesterol/amyloids interplay is enhanced in the cholesterol-rich brain [184], [185], and includes the interaction of cholesterol with tau [186], [187], prion [188], Aβ and [184], [189] and also plays a role in other amyloids such as IAPP and depends on the type and consistency of lipid membranes [123]. Measuring lipids alterations in lipid rafts is even suggested as a potential biomarker for neurodegenerative diseases. Interestingly, the amyloidogenic processing of APP takes place in lipid rafts while the non-amyloidogenic processing predominantly happens in the non-raft regions [184].

**Fig. 6:**
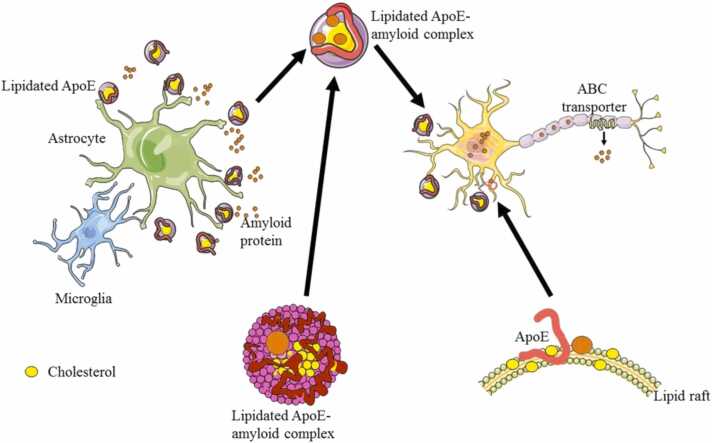
Secretion of lipidated ApoE by microglia and astrocytes and complex formation with extracellular and intracellular amyloid proteins.

ApoE, together with other apolipoproteins, lipoprotein receptors, and lipid transporters, is also important for the maintenance of cholesterol homeostasis in the brain [190]. Generally, ApoE is lipidated, and lipid association can enhance the affinity of ApoE for the LDL receptors via the lipid binding-induced conformational changes[191]. Lipidated ApoE is mostly produced by microglia and astrocytes, illustrated in Fig. 5. It can bind to soluble Aβ in an isoform-dependent manner (ApoE2 > ApoE3 > ApoE4), facilitating Aβ degradation via receptor-mediated endocytosis in various brain cells or clearance via the BBB [184]. In contrast to this differential binding affinity of lipidated ApoE for soluble Aβ, ApoE4 is less lipidated than E3 and E2. Furthermore, ApoE4 cannot efficiently protect neuronal cells by assisting liquid droplet formation in glia in response to elevated reactive oxygen species (ROS) [192], [193]. In addition, lipid-poor ApoE is more prone to cleavage and misfolding [3]. Together these factors indicate the intensive involvement of ApoE4 and increased risk in AD and another amyloidosis. However, the effect of cholesterol on the Aβ processing pathway may be both harmful and favorable, probably due to the promotion effect of high cholesterol levels within lipid rafts on Aβ generation (harmful) as well as degradation or clearance (favorable) [184], suggesting the importance of maintaining lipid homeostasis (Fig. 6).

As mentioned before, α-Syn potently stimulates cholesterol efflux in a dose- and time-dependent manner via ABCA1 transporter [194], which regulates the lipidation state of ApoE [195]. Tau pathology promotes the level of cellular unesterified cholesterol in neurons that is independent of apolipoprotein E deficiency. Synergically, a loss of cholesterol homeostasis may simulate tau pathology [186]. Obviously, interactions between cholesterol, ApoE and amyloid proteins like Aβ [196], tau [197] and α-Syn occur in various neurodegenerative disorders, and lipid rafts may provide an important site for this kind of interaction. However, whether lipid raft is a specific mechanism on how manipulation of cholesterol levels modifies APP/Aβ and whether cholesterol is a causal or non-causal factor for AD or both remains uncertain [198], since cholesterol may be both deleterious (when its homeostasis is perturbed) and favorable (when this homeostasis is maintained). A study in astrocyte-conditioned media suggested that ER–mitochondrial communication and MAM (mitochondria-associated ER membranes) function, which is responsible for lipid regulation and cholesterol trafficking, is upregulated with lipoproteins containing ApoE4 as compared to ApoE3, while no difference was observed with lipid-free ApoE [199]. Another study has reported that it is the transfer of cholesterol from the cytofacial leaflet to the exofacial leaflet (from inside to outside the cell), not the change in total cholesterol concentration, that promotes APP and Aβ expressions [200]. Besides, this transfer could disrupt membrane structure including lipid rafts and different kinds of cell functions including disruption in cholesterol homeostasis [200]. This is consistent with the fact that both increasing age and the inheritance of the ApoE4 allele can lead to the doubling of cholesterol levels in the exofacial leaflet [200]. On the other hand, cholesterol impacts the lateral organization of lipid membranes with condensation effect and therefore induces the interaction of amyloid protein and ApoE protein in lipid draft or lipidated complex. These suggest that lipid-related modulators, such as cholesterol, are involved in the interactions between ApoE and amyloid proteins, perhaps occurring in lipid raft and lipidated complex.

To explain the interactions of ApoE and amyloid proteins in the presence of lipids, a theoretical model can be used. Assuming a coupling, amyloid aggregates are stabilized through intramolecular and intermolecular interactions [201]. Intramolecular interaction occurs among amyloid or ApoE protein, due to their disordered and flexible conformations. These conformational states are affected by ordered alpha-helix/β-sheet and disordered turn/coil structures [202]. The conformational interchanges may minimize the total free energy, by coupling to intermolecular assembly. Once amyloid protein interacts with ApoE, the conformational interchanges and intermolecular assembly spontaneously emerge to reach a lower total free energy than ApoE or amyloid protein alone. Furthermore, a lipid-enriched environment converts the intramolecular conformations of ApoE and amyloid proteins with more ordered alpha-helix/β-sheet structures and lower energy [23], [203]. This may explain why amyloid protein interaction with ApoE happens and is prone to the lipid-related loci mentioned above.

## 5 Summary and perspectives

ApoE interacts with amyloid proteins in multiple ways, as described in this paper, and these interactions play an important role in the pathogenesis of several neurodegenerative diseases. Although not every person with ApoE4 develops neurodegenerative diseases and many patients with neurodegenerative diseases possess one of the other ApoE alleles, there clearly appears an increased risk for ApoE ε4 carriers. Therefore, the modulation of ApoE structure, expression, lipidation and function could be utilized for developing novel therapeutic approaches for degenerative diseases. More specifically, ApoE-based therapies are currently being investigated, mainly in the treatment for AD, with major focus on: a) the use of small molecule structural correctors that are able to convert the ApoE4 structure and function into those of ApoE3 [204], [205]; b) the overexpression of protective ApoE2, by adeno-associated virus delivery in brain [206] and the downregulation of ApoE4, by ApoE4 antisense oligonucleotides [207]; c) the inhibition of the binding between ApoE and Aβ, by using antibodies against Aβ targeting the ApoE binding site or antibodies against ApoE targeting the Aβ binding site [76]; d) increase of ApoE lipidation via ABCA1 [208]. It should be noted that the overexpression of ApoE2 may not be a reasonable approach as ApoE2 has been associated with the disorder type III hyperlipoproteinemia and with both increased and decreased risk for atherosclerosis and PD.

The study of ApoE and amyloid proteins and their roles in diseases proved extremely difficult mainly because the structures of these proteins are very heterogeneous and their aggregation and function are prone to profound changes under physiological and pathological conditions. Additionally, there are multiple in vivo components involved that are often left out in many studies of ApoE and amyloid proteins. Furthermore, many studies ignore or fail to report specific parameters, such as the lipidation, sialylation, oxidation or glycation state of ApoE, which makes it more difficult to compare results and draw consistent conclusions. Despite combining all the studies, we are still not able to conclude what is the isoform-dependent optimal range of ApoE concentration and lipidation state. However, we elaborated that the isoform-dependent lipidation state of ApoE and its intermediates affect the binding to cholesterol, which in turn differently affects the production, stabilization, degradation, clearance, binding and transport of different amyloids in various aggregation states (monomers, oligomers or fibrils). As one of the strongest genetic risk factors for neurodegenerative diseases, ApoE4 affects normal brain function in mice and humans long before obvious pathological changes are noticeable [209]. ApoE4 is less stable and (its fragments) more toxic/less protective in comparison with ApoE2 and ApoE3, although more protective/less toxic compared to the ApoE knocked-out mice. Because not every person with ApoE4 develops diseases, it is most likely that all ApoE isoforms are highly functional within homeostasis. The detrimental effects of ApoE can be subscribed to either the loss of protective function or the gain of toxic effect under disturbed and physiologically stressed conditions. And this change in functions of ApoE will occur when changes in the concentration, morphology and/or modification levels of ApoE are too dramatic. Besides, it is probable that compared to ApoE3 and ApoE2, ApoE4 seems more susceptible and shows a stronger negative response to an induced loss of homeostasis. ApoE4 is prone to formation of high entropic and partially folded structures [17]. This enhanced susceptibility of ApoE4 can be induced mainly by other components, like amyloid proteins and can be considered as an important factor for the understanding of neurodegeneration as well as the development of potential therapeutics.

Why there occurs a loss of homeostasis in amyloid diseases in the first place remains to be further investigated. There is an increasing awareness that neurodegenerative disorders including AD and PD are multifactorial diseases [210], [211]. The majority of complex and chronic diseases are the result of the interplay between multiple (risk) factors, which can be categorized in genetic, environmental and lifestyle factors. Together they influence the microscopic and dynamic environment of cells and their (dys)function, including the (mal)functioning of the identified target molecules (amyloid proteins) related to specific diseases. This awareness has resulted in an increasing interest in systems biology, personalized medicine and multimodal therapy [212], because any therapy for multifactorial diseases that focuses only on a single factor has a low chance of success. We therefore suggest that some of the aforementioned ApoE-based therapies might be more successful when combined with other therapies or by addressing other known risk factors (e.g., endogenous factors) that are most likely being ignored in monotherapies.

ApoE-based therapies for amyloidosis show great therapeutic potential in this field, especially after the failure of therapies by targeting only amyloids. However, developing an ApoE-based therapy targeting only the pathology-related function is challenging. On the one hand, ApoE has various functions, on the other hand, ApoE is closely interwoven with amyloid proteins involved in various amyloidosis. Even though confronted with challenges, there are important points that can be taken into account when developing ApoE-based therapies. Firstly, try to understand as much as possible the exact normal functions of ApoE and their roles in amyloidosis, and to restore the balance of ApoE. Secondly, learn more about the cross-interactions between ApoE and amyloids and the stress conditions that ApoE is involved in, which is the main purpose of this review. Thirdly, recognize the discrepancies in the conditions of the in vitro and in vivo studies, as well as the differences between animal models and clinical trials, when interpreting the investigation results. Otherwise, the intended therapeutic effects may not be achieved and unintended negative consequences might occur. Moreover, the connection between ApoE/amyloid proteins and chronic inflammation, oxidative stress and metal homeostasis [16], [78], [124] as well as the microbiome–gut–brain axis (which is also ApoE-dependent) are strongly associated with neurodegenerative diseases [213], which are other complex factors in most diseases that go beyond the scope of this paper.

## CRediT authorship contribution statement

**Rolf Antonie Loch:** Writing – original draft. **Hongzhi Wang:** Writing – original draft. **Alex Perálvarez Marín**: Writing – review & editing. **Philipp Berger:** Writing – review & editing. **Henrietta Nielsen:** Writing – review & editing. **Angeliki Chroni:** Writing – review & editing. **Jinghui Luo:** Conceptualization, Investigation, Supervision, Writing – review & editing.

## Declaration of Competing Interest

The authors declare that they have no known competing financial interests or personal relationships that could have appeared to influence the work reported in this paper.
